# Oxford Nanopore Technologies Sequencing and Targeted Amino Acid Metabolomics Reveal Spatially Segregated Microbial Hijacking and Metabolic Collapse During *Trichoderma* Infection of Golden Ear Mushroom

**DOI:** 10.3390/foods15111912

**Published:** 2026-05-28

**Authors:** Yijing Xu, Yu Huang, Lei Ye, Jiang Yu, Zhengzhu Huang, Xuezhen Yang, Qing Tian, Bo Zhang, Yuntao Liu, Xiaolin Li

**Affiliations:** 1 Sichuan Institute of Edible Fungi, Sichuan Academy of Agricultural Sciences, Chengdu 610066, China; yijinxu7@gmail.com (Y.X.); huang_yu@scsaas.cn (Y.H.); yeray@scsaas.cn (L.Y.); huangzhenzhu123@scsaas.cn (Z.H.); yangxz1986@scsaas.cn (X.Y.); tianqing230909@163.com (Q.T.); zhangbo1987@scsaas.cn (B.Z.); 2 Key Laboratory of Coarse Cereal Processing, Ministry of Agriculture and Rural Affairs, Sichuan Engineering & Technology Research Center of Coarse Cereal Industrialization, School of Food and Biological Engineering, Chengdu University, Chengdu 610106, China; 3 Sichuan Xinzhongyu Agriculture Co., Ltd., Suining 629111, China; 13133117280@163.com; 4 College of Food Science, Sichuan Agricultural University, Chengdu 611134, China; liuyt@sicau.edu.cn

**Keywords:** *Naematelia sinensis*, *Trichoderma* infection, microbial community, amino acid metabolism, Oxford Nanopore sequencing

## Abstract

This study combines Oxford Nanopore (ONT) third-generation sequencing with targeted amino acid metabolomics to elucidate the mechanisms underlying the structural and metabolic responses of the microbial community in Golden Ear Mushroom (*Naematelia sinensis*) during *Trichoderma* infection. By comparing healthy tissue (MOCK), adjacent healthy areas (HAF) and the core lesion area (DiR), the results indicate that pathogen infection significantly reduces bacterial community diversity, with a progressive decline observed across these regions. In the DiR region, the fungal community underwent significant restructuring, with the abundance of the *Trichoderma* genus (*T. lixii* and *T. afroharzianum*) rising to over 45%, whilst that of host symbiotic fungi (*Stereum* and *Tremella*) decreased by 50–60%. Metabolomic analysis indicated that levels of various amino acids and antioxidant-related metabolites were significantly reduced in the host tissue of the DiR region, suggesting that amino acid metabolism was inhibited. Concurrently, changes were observed in certain metabolites associated with nitrogen metabolism (e.g., L-glutamine). KEGG analysis further revealed that amino acid biosynthesis and D-amino acid metabolic pathways were inhibited, whilst ABC transporters and arginine/proline metabolic pathways were activated. All metabolic changes originated from the host fungal tissue itself, rather than from commensal microorganisms. In summary, *Trichoderma* may promote the infection process by disrupting the host microbial community and metabolic networks, providing a theoretical basis for understanding the mechanisms of fungal diseases and their control.

## 1. Introduction

Golden Ear Mushroom (*N. sinensis*), named for its yellow fruiting bodies, is a significant edible and medicinal fungus in China that has achieved large-scale commercial cultivation in recent years [1]. Research confirms that the species currently cultivated extensively in China is *N. sinensis*, not *Tremella aurantialba* or *N. aurantialba* as previously misidentified [2]. Furthermore, *N. sinensis* is not a single species but forms a stable symbiotic system with *Stereum hirsutum* [3].

*N. sinensis* holds significant ecological and economic value, with its medicinal properties extensively studied. These properties confer significant application potential in traditional medicine [4] and modern drug development [5,6,7].

Despite its numerous advantages, *N. sinensis* is notably susceptible to fungal infections. For example, the initial documented instance of brown rot disease, attributed to *Ewingella americana*, was identified at a farm in Tongzhou District, Beijing. This disease induces a color transformation in the fruiting bodies from orange to light brown, eventually leading to shriveling and browning, which significantly diminishes yield and results in economic losses [8]. Similar disease-associated symptoms and physiological deterioration have also been reported in other edible fungal cultivation systems, indicating that cultivated mushrooms are highly vulnerable to opportunistic microbial infections under intensive production conditions [9]. These cases underscore a prevalent susceptibility to fungal diseases among edible mushrooms and other critical crops, highlighting the imperative for further research to develop effective control strategies.

*Trichoderma* species, well-known biocontrol agents, are widely used to combat plant pathogenic fungi [10], though they also have the potential to act as opportunistic pathogens. *Trichoderma* fungi infect their hosts through a variety of mechanisms, including enzymatic degradation and toxin secretion. Studies have shown that *T. harzianum* invades host tissues by secreting cellulases and chitinases that degrade the host cell wall [11]. Additionally, *Trichoderma* species produce secondary metabolites such as harzianum A and aspinolides, which suppress the growth of other pathogenic fungi [12]. In edible mushroom cultivation, *Trichoderma* infections can lead to brown spot disease, significantly reducing both yield and quality. Due to their rapid growth and strong competitive advantage in nutrient acquisition, *Trichoderma* fungi can quickly dominate the growth environment of edible fungi, inhibiting their development. Furthermore, the toxins they secrete can disrupt cellular structures and metabolic processes, further diminishing productivity [13]. These findings emphasize the dual nature of *Trichoderma* species as both beneficial biocontrol agents and potential pathogens. Understanding their interactions with edible fungi is crucial for developing effective strategies to mitigate disease outbreaks and ensure sustainable mushroom production.

Despite recent advancements in the study of pathogen-edible mushroom interactions, the majority of research remains limited to single-domain interactions. Investigations into the bioactivity of edible mushrooms have predominantly concentrated on their antioxidant, antihypertensive, and antimicrobial properties [14]. Similarly, although fungal–bacterial interactions have been demonstrated to exhibit both synergistic and antagonistic relationships [15], the mechanisms underpinning edible mushroom-pathogen interactions remain largely unexplored. Specifically, research on *Trichoderma*-host interactions has primarily addressed either bacterial or fungal communities in isolation, lacking a comprehensive analysis of cross-domain interactions. This limitation is not exclusive to mushroom research but is also prevalent in studies of plant and animal microbiomes. Increasing evidence indicates that bacterial-fungal interactions play a pivotal role in shaping host ecological niches, where microbial imbalances may contribute to disease onset [16].

Amino acids and their metabolites serve as crucial mediators in host–microbe interactions, particularly concerning host stress responses. Beyond their involvement in immune modulation, amino acid metabolism plays a central role in signaling pathways and antioxidant defense mechanisms. For example, metabolites derived from aromatic amino acids are instrumental in regulating immune, metabolic, and neurological responses, with disruptions in these chemical interactions being linked to gastrointestinal and systemic diseases [17]. Under conditions of fungal infection response, amino acid metabolism undergoes significant alterations: biosynthesis is downregulated during energy depletion, while amino acid-based respiratory pathways are activated as alternative energy sources—an adaptive strategy essential for metabolic adaptation under adverse conditions [18]. Furthermore, pathogens can manipulate amino acid metabolism to modulate host immunity and enhance their own survival [19].

Traditional single-omics methodologies are inadequate for deciphering the intricate biological interactions inherent in complex systems. Recent advancements in third-generation sequencing technologies, exemplified by ONT, in conjunction with targeted metabolomics, provide unparalleled opportunities to elucidate the multifaceted “microbe–metabolite–host” interactions. Third-generation sequencing offers high-throughput, long-read genomic data, which facilitates comprehensive profiling of microbial communities. The integration of genomic and metabolomic datasets enhances the precision of our understanding of microbial metabolic pathways and their consequential effects on host physiology. Additionally, targeted metabolomics allows for the accurate quantification of specific metabolites, thereby illuminating the metabolic interactions between microbes and hosts. This integrative approach not only aids in the identification of metabolic markers associated with diseases but also clarifies the functional roles of microbial metabolites in host physiological processes.

Although *N. sinensis* is widely recognised as forming a stable symbiotic relationship with *Stereum hirsutum*, the mechanism by which *Trichoderma* infection disrupts this fungal association remains unclear. Given that the genus *Trichoderma* is known to be a fungal parasite capable of entangling, penetrating and degrading the cell walls of host fungi through the secretion of chitinases and glucanases [20], we hypothesise that the *Trichoderma* strain used in this study may have directly attacked the hyphae of S. hirsutum, thereby physically disrupting the symbiotic interface between *N. sinensis* and *S. hirsutum.* Furthermore, *Trichoderma* may indirectly disrupt the nutritional exchange basis of this symbiotic system by secreting antimicrobial secondary metabolites (such as peptidols and ketones) that inhibit the growth of *S. hirsutum* [21]. It is worth noting that previous reports have indicated that *S. hirsutum* and related species of the genus Stereaceae serve as hosts for various parasitic ascomycetes within the order Hypocreales, an order which also includes the genus *Trichoderma* [22]; this provides an ecological precedent for such a three-way interaction. Verification of this hypothesis would aid in identifying molecular targets for *Trichoderma* antagonism within a symbiotic context and provide new insights into tripartite fungal interactions within complex microbial communities.

In light of the aforementioned context, this study seeks to explore two pivotal research questions: (1) In what manner does *Trichoderma* infection alter the cross-domain microbial community, specifically the bacteria–fungi interactions, within *N. sinensis*? (2) How do principal microbial taxa and amino acid metabolites collaboratively influence disease progression and host defense mechanisms? Employing a multi-omics methodology, we aim to elucidate the processes by which *Trichoderma* infection induces restructuring of the cross-domain microbiome in *N. sinensis* and to analyze the coordinated regulation of metabolic pathways, including amino acid metabolism and antioxidant defense. Our research is anticipated to uncover the mechanisms underlying microbial “hijacking” and host metabolic reprogramming in response to *Trichoderma* infection, thereby identifying theoretical targets—such as key pathogenic species or protective metabolites—for the development of microbiome-based biocontrol strategies. This study is expected to provide valuable insights into disease mitigation strategies aimed at counteracting the yield and quality losses in *N. sinensis* production induced by *Trichoderma*.

## 2. Materials and Methods

### 2.1. Environmental Comparison and Sample Collection

**Figure 1:**
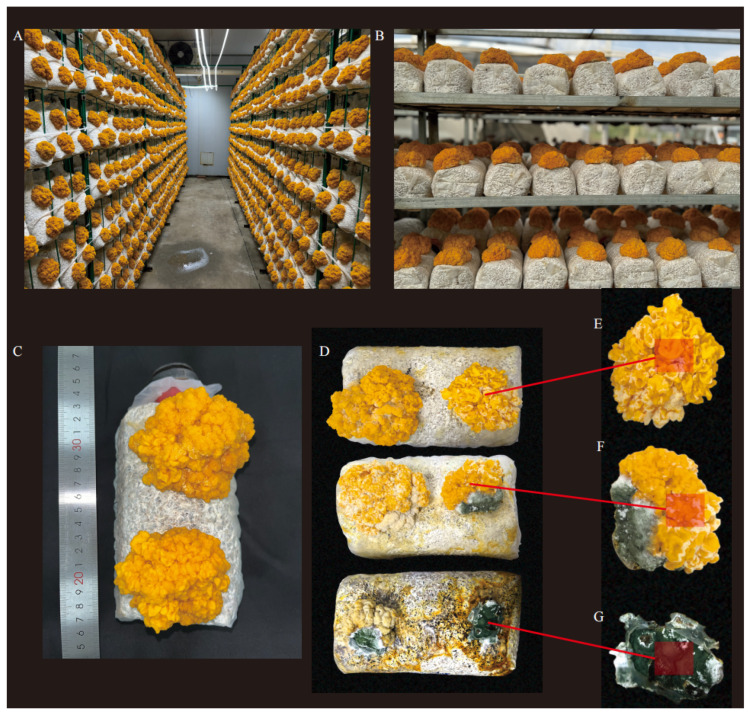
Cultivation Models of *N. sinensis* and Experimental Sample Characterization. (**A**) Industrial cultivation scene of *N. sinensis*, displaying a standardized environmental control system. (**B**) Comparison of traditional greenhouse cultivation environments for *N. sinensis*. (**C**) Healthy mushroom bags in industrial cultivation (intact substrate, even distribution of mycelium). (**D**) Morphological comparison of fungal bags under three treatments (top to bottom: MOCK, HAF, DiR). (**E**) MOCK group sample, healthy *N. sinensis* tissue. (**F**) HAF group sample, pathogen infection boundary with seemingly healthy tissue (within 2 cm of the disease site). (**G**) DiR group sample, brown lesions caused by *Trichoderma* spp. infection.

A comparative analysis of environmental conditions between the industrial cultivation system (Figure 1A) and the traditional cultivation model (Figure 1B) revealed a higher susceptibility to infection in the industrialized system. Systematic monitoring of pathological browning in fungal cultivation bags from a commercial production facility (Figure 1C vs. Figure 1D) facilitated the design of a three-tiered treatment group (Figure 1D). The MOCK group (healthy control) consisted of fully developed *N. sinensis* fruiting bodies with evenly distributed mycelia, randomly sampled from disease-free bags (Figure 1E). The HAF group included transitional tissue excised 2 cm from the periphery of *Trichoderma* infection sites, which exhibited no visible symptoms (Figure 1F). The DiR group consisted of tissue samples precisely extracted from the central browning zone, characterized by a dark brown discoloration and soft, decayed texture, serving as representative diseased specimens (Figure 1G). All samples (*n* = 3 per group) were collected from standardized cultivation bags within the same production batch and were prepared and processed independently to ensure consistency.

### 2.2. DNA Extraction and Sequencing

Genomic DNA was isolated from *N. sinensis* specimens utilizing the OMEGA DNA Kit (M5635-02, Omega Bio-Tek, Norcross, GA, USA) in accordance with the manufacturer’s instructions. The isolated DNA was subsequently stored at −20 °C until further analysis. The concentration and quality of the DNA were evaluated using a NanoDrop NC2000 spectrophotometer (Thermo Fisher Scientific Inc., Waltham, MA, USA) and verified through agarose gel electrophoresis.

Full-length 16S rRNA gene amplification was performed using the forward primer 27F (5′-AGAGTTTGATCMTGGCTCAG-3′) and the reverse primer 1492R (5′-ACCTTGTTACGACTT-3′) [23]. For fungal identification, full-length ITS gene amplification was conducted using the forward primer ITS1F (5′-CTTGGTCATTTAGAGGAAGTAA-3′) and the reverse primer LR3 (5′-CCGTGTTTCAAGACGGG-3′) [24]. Amplicon sequencing of the 16S rRNA gene was performed following the methodology described by Yang et al. (2018) [23], while fungal ITS gene amplicon sequencing was conducted according to Loit et al. (2019) [25].

### 2.3. Sequence Analysis

Nanopore sequencing was performed on the ONT PromethION P48 platform. Library preparation was carried out using the SQK-LSK109 ligation sequencing kit (Oxford Nanopore Technologies, Oxford, UK), in accordance with the manufacturer’s instructions. DNA concentration was quantified using a Qubit 4.0 fluorometer (Thermo Fisher Scientific, Waltham, MA, USA), and integrity was assessed via 0.35% agarose gel electrophoresis. Sequencing was performed using R10.4.1 flow cells. Base calling and barcode demultiplexing were performed using Guppy v5.0.16 in high-accuracy mode (HAC); low-quality reads were optionally re-called using super-accuracy mode (SAC) to improve base-level precision. Raw reads were quality-filtered (minimum Q ≥ 7) and length-filtered (minimum 500 bp) using NanoFilt (https://github.com/wdecoster/nanofilt, accessed on 13 February 2025). Putative chimeric sequences were identified and removed using VSEARCH (https://github.com/torognes/vsearch, accessed on 13 February 2025) before downstream taxonomic annotation.

Given the characteristics of ONT sequencing (e.g., long read lengths but a relatively high single-base error rate), an alignment-based approach was adopted for amplicon analysis. Specifically, the LAST software (https://gitlab.com/mcfrith/last, accessed on 13 February 2025) was used to align sequences against reference databases. Taxonomic annotation of bacterial 16S rRNA gene sequences utilised the Silva database (version 138) [26], whilst fungal ITS sequences were annotated using the UNITE database (version 8.2) [27]. For each sequence, the alignment result with the highest score was selected as the best match and used to construct a feature table. Once the feature table was generated, QIIME2 [28] (version 2022.11, analysis followed the official QIIME2 pipeline (https://docs.qiime2.org/2022.11/tutorials/, accessed on 8 February 2025) was used to filter out low-abundance features. All downstream analyses followed the official QIIME2 workflow.

### 2.4. Sample Preparation and Extraction for UPLC-MS/MS Analysis

After thawing and homogenization, 0.05 g of each sample was combined with 500 µL of a 70% methanol/water solution. The mixture was vortexed for 3 min at 600× *g* and subsequently centrifuged at 13,800× *g* for 10 min at 4 °C. Following centrifugation, 300 μL of the supernatant was transferred to a new centrifuge tube, stored at −20 °C for 30 min, and centrifuged again at 13,800× *g* for 10 min at 4 °C. Thereafter, 200 μL of the supernatant was extracted using a Protein Precipitation Plate for subsequent LC-MS analysis.

### 2.5. UPLC-MS/MS Analysis for Targeted Metabolomics

The sample extracts were analyzed using an LC-ESI-MS/MS system (UPLC, ExionLC AD; MS, QTRAP^®^ 6500+ System) from SCIEX (Framingham, MA, USA). Chemical reagents followed Ye et al. (2024) [29]. The analysis used an ACQUITY BEH Amide HPLC column (2.1 × 100 mm, 1.7 μm) with a solvent system of water and acetonitrile, both containing 2 mM ammonium acetate and 0.04% formic acid. The gradient was 90% B (0–1.2 min), 60% B (9 min), 40% B (10–11 min), then back to 90% B (11.01–15 min). The flow rate was 0.4 mL/min, column temperature 40 °C, and injection volume 2 μL.

The AB 6500+ QTRAP^®^ LC-MS/MS System was equipped with an ESI Turbo Ion-Spray interface, operating in both positive and negative ion modes, controlled by Analyst 1.6 software (AB Sciex, Framingham, MA, USA). The ESI source parameters were as follows: ion source, turbo spray; source temperature, 550 °C; ion spray voltage (IS), 5500 V (positive) and −4500 V (negative); curtain gas (CUR), 35 psi. DP and CE for individual MRM transitions were optimized accordingly. Specific MRM transitions were monitored based on the amino acids eluted within each period.

### 2.6. Bioinformatics and Statistical Analysis

Sequence data were primarily analyzed using QIIME2 and R packages (version 4.2.1). Based on the amplicon sequence variant (ASV) table, QIIME2 was used to calculate alpha diversity indices, including ACE and Chao-1 abundance estimates, as well as Shannon and Simpson diversity indices. The following tests were performed for statistical comparisons among the three groups (MOCK, HAF, DiR). Alpha diversity indices were compared overall using one-way ANOVA, followed by pairwise comparisons using Tukey’s Honest Significant Difference (HSD) post hoc test. Beta diversity (assessed based on Bray–Curtis and Jaccard distance matrices) was analyzed using PERMANOVA (adonis2 function) with 999 permutations, with group (MOCK/HAF/DiR) as the main factor. Unless otherwise specified, all results are presented as mean ± standard deviation.

LEfSe (Linear Discriminant Analysis Effect) analysis was used to identify species with statistically significant differences between groups. Parameter settings were as follows: LDA score (log10) threshold: ≥4 for fungal ITS data and ≥2 for bacterial 16S data; Kruskal–Wallis test with a significance level of α = 0.05 was used for preliminary feature screening; Paired Wilcoxon test with α = 0.05 was used for intergroup comparisons, employing a strategy of pairwise comparisons among all groups. Visualizations generated by LEfSe, including LDA score distribution plots, phylogenetic trees, PCA plots, clustering heatmaps, and differential metabolite violin plots, were created using R packages; other figures were generated using Origin 2021 (OriginLab, Northampton, MA, USA). To control the false positive rate resulting from multiple comparisons, *p*-values were corrected using the false discovery rate (FDR) based on the Benjamini–Hochberg method. Corrected *p*-values (q-values) < 0.05 were considered statistically significant.

Metabolite annotation was performed using the KEGG Compound Database (http://www.kegg.jp/kegg/compound/, accessed on 23 February 2025) and mapped to the KEGG Pathway Database (http://www.kegg.jp/kegg/pathway.html, accessed on 20 February 2025). Metabolite set enrichment analysis (MSEA) was conducted on pathways containing significantly differentially expressed metabolites, and statistical significance was determined by the *p*-value from the hypergeometric test.

## 3. Results

### 3.1. Oxford Nanopore Technology Analysis

#### 3.1.1. Alpha Diversity Analysis

**Figure 2:**
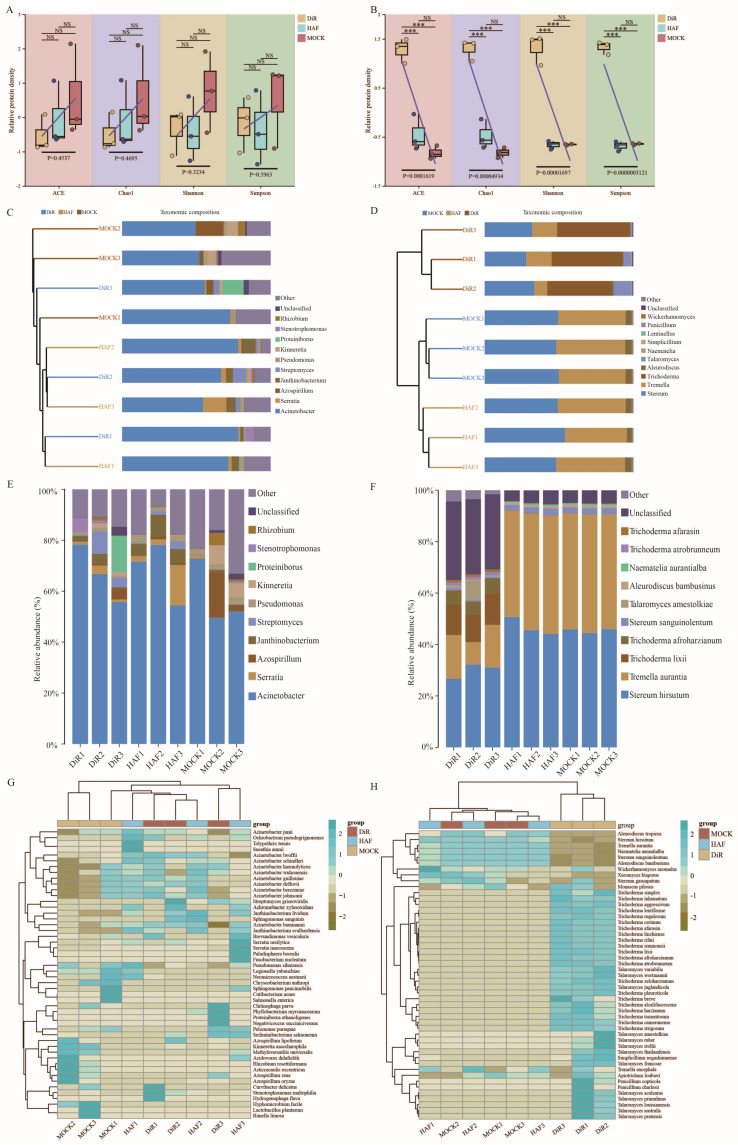
Microbial Diversity and Community Structure Analysis. (**A**) Comparison of bacterial α-diversity indices between groups, with the blue regression line equation: y = a + bx; NS indicates no significant difference (same for all subsequent figures). (**B**) Fungal α-diversity indices, *** indicates significant differences between groups (*p* < 0.001, ANOVA). (**C**,**D**) Bacterial (**C**) and fungal (**D**) community UPGMA clustering trees based on the binary Jaccard algorithm (left), with branch length reflecting sample similarity; bar plots on the right show relative abundance of top 10 genera at the genus level. (**E**,**F**) Bacterial (**E**) and fungal (**F**) species-level composition, showing only the top 10 species, with “Others” representing low-abundance groups and “Unclassified” indicating unannotated species. (**G**,**H**) Z-score standardized abundance heatmap for bacterial (**G**) and fungal (**H**) species-level data (blue: high abundance, green: low abundance).

The examination of alpha diversity indices, standardized using z-scores (Figure 2A), in conjunction with raw data (Table S1), revealed a distinct gradient of bacterial community infection among the MOCK (healthy tissue), DiR (diseased area), and HAF (adjacent healthy zone) groups. At the diseased site (DiR), several diversity indices were significantly reduced compared to the healthy tissue (MOCK), with notable declines observed in both the ACE and Chao1 indices. The standard deviation of the Shannon index in the DiR group (SD = 0.38) was substantially lower than that of the MOCK group (SD = 0.57), suggesting a decrease in bacterial community evenness and a more homogeneous structure at the disease site. The HAF group exhibited an intermediate profile, characterized by a wide range of Shannon indices (3.04–4.16) and a Simpson index (0.76 ± 0.07) that was lower than that of the MOCK group (0.80 ± 0.07). Nonetheless, some indices did not display significant differences (*p* > 0.05), indicating that while the bacterial community in the adjacent region may be indirectly influenced by pathogen spread, it still retains relatively stable community characteristics. The MOCK group exhibited optimal characteristics across all indices, reflecting higher bacterial richness and community stability in healthy tissue.

The fungal community response to infection differed significantly from that of the bacteria (Figure 2B, Table S2), with a sharp drop in diversity in the DiR group. Diversity in the DiR group was notably lower than in both the MOCK and HAF groups, especially in terms of Shannon and Simpson indices (*p* < 0.001). The Shannon index (3.38 ± 0.52) and Simpson index (0.76 ± 0.07) of the DiR group were lower than those in the MOCK group (Shannon 3.98 ± 0.78, Simpson 0.80 ± 0.07), suggesting that pathogen infection resulted in a decrease in fungal diversity and evenness. Although the HAF group had lower diversity than the MOCK group (Shannon 3.69 ± 0.54, Simpson 0.79 ± 0.07), the differences were not significant (NS), potentially due to host defense mechanisms or spatial isolation, indicating that the adjacent region may have maintained higher fungal diversity through some regulatory mechanisms. The ACE and Chao1 richness indices indicated that the MOCK group had significantly higher values than the DiR group, with the HAF group showing intermediate values, indicating that pathogen infection significantly impacted fungal species richness.

Bacterial diversity showed a continuous decrease along the infection gradient (MOCK → HAF → DiR), while fungal diversity experienced a sharp loss only in the infected area. Key data indicated that healthy tissue (MOCK) established a disease-resistant microenvironment by maintaining high richness (bacterial ACE > 268, fungal ACE > 315) and high evenness (bacterial Shannon > 4.24, fungal Shannon > 3.98). Furthermore, compared with the other groups, bacterial heterogeneity was observed in the HAF group (SD > 0.5), whilst fungal diversity remained relatively stable across samples.

#### 3.1.2. Beta Diversity Analysis

Beta diversity analysis was performed using the QIIME2 package to assess the similarity of species diversity between different samples. The distance between samples was calculated using the binary Jaccard algorithm to obtain the β-values. Based on the Beta diversity analysis (binary Jaccard algorithm) and species composition data, significant differences were observed in the bacterial and fungal communities across different treatments (MOCK, DiR, HAF), with patterns closely linked to the dynamics of key microorganisms (Figure 2C,D). *Trichoderma* infection triggered a cascade of reactive changes in both bacterial and fungal communities, with bacterial communities transitioning from a stable symbiotic state in healthy tissue to a disrupted state in the diseased region, while fungal communities shifted from a state of pathogen resistance to an imbalanced condition at the disease site.

For the bacterial community (Figure 2C), the MOCK samples exhibited the smallest distances between them (e.g., MOCK1-MOCK2 Jaccard = 0.801), indicating a stable community structure dominated by Acinetobacter, with co-occurring symbionts such as *Azospirillum*, *Kinneretia*, *Rhizobium*, *Pseudomonas*, and *Stenotrophomonas*. This stable bacterial composition likely reflects a mutualistic host-microbe relationship in a healthy state. In the DiR, the Jaccard distance significantly increased (e.g., DiR1-MOCK2 = 0.843), and bacterial diversity declined, with the community structure becoming notably disrupted: beneficial bacteria like Rhizobium decreased in abundance, while pathogenic bacteria such as *Proteiniborus* increased, and the proportion of uncategorized microbes rose. These changes suggest that *Trichoderma* infection severely disturbed the bacterial community, potentially weakening the host’s defense mechanisms against pathogens. Interestingly, although the HAF appeared normal, its microbial community exhibited early response features: the distance to MOCK increased significantly (e.g., HAF1-MOCK2 = 0.825), with partial overlap with the DiR group (e.g., HAF3-DiR3 = 0.763). This phenomenon may indicate that specific genera such as *Janthinobacterium*, *Streptomyces*, and *Proteiniborus* play important roles in the host’s early defense response.

Regarding the fungal community (Figure 2D), *Trichoderma* infection also caused significant stepwise differentiation. The MOCK group samples had the smallest distances between them (e.g., MOCK2-MOCK3 Jaccard = 0.381), and the fungal community structure was stable, with *Stereum*, *Tremella*, and *Aleurodiscus* as the dominant taxa (accounting for >90%). Pathogenic *Trichoderma* was almost undetectable, suggesting that healthy fungal communities are capable of effectively resisting pathogen colonization. In contrast, the Jaccard distance in the DiR group significantly increased (DiR3-MOCK2 = 0.761), and intra-group heterogeneity also increased (DiR1-DiR2 = 0.354), with *Trichoderma* representing over 45% relative abundance, directly confirming its pathogenic role. Furthermore, the increase in saprophytic fungi such as *Talaromyces* suggests that *Trichoderma* infection not only disrupted fungal community balance but might also accelerate tissue decay. Notably, the HAF group, though appearing healthy, showed a significantly smaller distance to the DiR group (e.g., HAF3-DiR3 = 0.708) compared to the MOCK group (HAF3-MOCK2 = 0.448), and the relative abundance of *Simplicillium* and *Talaromyces* increased. This phenomenon suggests that the adjacent healthy tissue may have recruited antagonistic microorganisms (e.g., *Simplicillium*) to form a “buffer zone” mechanism, limiting the further spread of the pathogen.

#### 3.1.3. Bar Chart Analysis of Species Composition at Different Taxonomic Levels

The bacterial communities of *N. sinensis* in the *Trichoderma*-infected treatment groups (DiR and HAF) exhibited significant differences from the control group (MOCK) at the phylum, genus, and species levels. At the phylum level (Figure S1A), *Proteobacteria* dominated all samples (average relative abundance > 85.6%); however, its abundance significantly decreased in the DiR group (e.g., DiR3: 68.7%), while Firmicutes abnormally increased to 22.6% in DiR3, far exceeding other samples (average: 0.8–6.8%). This suggests that pathogenic infection may stimulate the proliferation of specific Gram-positive bacteria. *Actinobacteriota*, with a low abundance in the MOCK group (<3.4%), exhibited a significant increase in DiR2, suggesting a potential role in host immune regulation. The highest abundance of Bacteroidetes was detected in MOCK3 (6.2%), potentially linked to the metabolic functions of healthy tissues. At the genus level (Figure S1C), *Acinetobacter* was the predominant genus across all samples (49.6–78.3%), showing increased abundance in DiR and HAF, possibly related to pathogen tolerance. *Serratia* displayed a sharp increase in HAF3 (15.9%), potentially associated with immune regulation near the infection site. Notably, *Proteiniborus* was exclusively detected in DiR3 (14.3%), a genus related to lignocellulose degradation, likely resulting from polysaccharide substrate release due to pathogen-induced tissue necrosis, making it a potential biomarker for pathogenic infection. *Pseudomonas* remained stable in the MOCK group (2.0–2.5%) but decreased to 0.6% in DiR3, indicating its sensitivity to environmental disturbances. At the species level (Figure 2E), *Acinetobacter guillouiae* was identified as a core species across all groups (DiR: 37.9–51.0%; HAF: 37.3–53.6%; MOCK: 32.1–51.4%), suggesting its foundational role in host microbiome stability. *Semarcescens* exhibited a dramatic increase in HAF3 (10.9%), possibly participating in host–pathogen interactions through quorum sensing. *Streptomyces griseoviridis*, abundant in DiR2 (6.2%), might contribute to pathogen suppression due to its antibiotic synthesis capability. The specific species *Proteiniborus* ethanoligenes was exclusively detected in DiR3 (14.3%), indicating its proliferation in response to metabolic byproducts at the infection site. Unclassified sequences reached 9.9% in MOCK3, reflecting the incomplete characterization of the healthy tissue microbiome.

Similarly, fungal community composition varied among groups. At the phylum level (Figure S1B), *Basidiomycota* dominated the MOCK group (99.7–99.9%) but significantly declined in DiR samples (43.3–49.8%), while *Ascomycota* increased dramatically (DiR1: 53.4%; DiR2: 56.6%; DiR3: 50.1%), indicating a structural shift driven by *Trichoderma* infection. In contrast, *Basidiomycota* abundance in HAF samples recovered to 99.7–99.8%, suggesting localized immune restoration. At the genus level (Figure S1D), *Trichoderma* was uniquely enriched in DiR (43.6–48.7%) but nearly absent in MOCK and HAF (<0.2%), confirming its role as a key pathogenic driver. *Stereum* and *Tremella* were highly abundant in MOCK (44.8–50.0%) but decreased in DiR (28.0–33.3% and 8.8–17.2%, respectively), likely due to competition with the pathogen. *Talaromyces* increased abnormally in DiR2 (12.2%), potentially assisting *Trichoderma* colonization via metabolic interactions. At the species level (Figure 2F), *T. lixii* and *T. afroharzianum* were predominant in DiR (10.7–12.2% and 5.1–5.9%, respectively) but negligible in other groups, highlighting their pathogenic roles. *Talaromyces amestolkiae* was prominent in DiR2 (7.6%), likely facilitating niche occupation during early infection. *S. hirsutum* remained stable in MOCK (44.4–45.8%) but declined in DiR (26.7–32.2%), reflecting pathogen-induced disruption of symbiotic functions. *T. aurantialba* increased in HAF (41.5–46.1%), potentially contributing to antimicrobial defense in adjacent tissues. Unclassified species in DiR averaged 29.7%, suggesting pathogen-induced complexity and the proliferation of yet-to-be-characterized species.

#### 3.1.4. Cluster Analysis of Species Abundance Heatmaps

The heatmap analysis of bacterial communities at different taxonomic levels revealed significant variations in bacterial composition among different treatment groups of *N. sinensis* infected by *Trichoderma*, particularly at the species level, where distinct pathogen–host interactions were observed. At the phylum level (Figure S1E), *Proteobacteria* dominated all three groups (DiR: 84.4%, HAF: 91.3%, MOCK: 87.9%), with a slightly higher abundance in HAF. Firmicutes exhibited a significant increase in DiR (8.4% vs. HAF 1.1% and MOCK 2.9%), and Bacteroidetes showed the highest abundance in MOCK (3.6%). Meanwhile, *Actinobacteriota* exhibited the lowest abundance in the DiR group.

At the genus (Figure S1G) and species levels (Figure 2G), dynamic shifts in pathogenic and beneficial bacteria were observed. *Pseudomonas* showed a significant increase in DiR (mean: 1.0% vs. MOCK: 2.2%), potentially exacerbating tissue damage through the secretion of secondary metabolites. *Acinetobacter* was more abundant in MOCK (mean: 58.2% vs. DiR: 66.9%), yet some pathogenic species, such as *A. baumannii*, were enriched in DiR. Additionally, *Legionella* and *Salmonella* were detected exclusively in DiR, whereas the symbiotic bacterium Candidatus *Saccharimonas* was specific to MOCK, highlighting the unique microbial barrier function of healthy tissues. At the species level, *A. guillouiae* and *A. lwoffii* were consistently present across all groups. *A. baumannii* increased in DiR (0.75–1.05%), with its multidrug resistance potentially exacerbating tissue damage. In HAF, *Pseudomonas sinuensis* exhibited a relatively higher abundance (1.1%), suggesting an activated defense response in adjacent tissues. In the healthy MOCK group, *Lactobacillus plantarum* and *Sediminibacterium salmoneum* were more abundant, likely contributing to microbial homeostasis through competitive pathogen inhibition. In HAF, *J. lividum* and *Rhizobium rosettiformans* were enriched, potentially mitigating disease spread through antimicrobial peptide secretion or host immune modulation.

In terms of fungal composition at the phylum level (Figure S1F), Basidiomycota overwhelmingly dominated the healthy MOCK group (>99.9%), while Ascomycota remained at a minimal proportion (<0.1%). However, in the infected DiR group, Ascomycota significantly increased to 50.1–56.6%, primarily driven by *Trichoderma* (43.6–48.7%), whereas Basidiomycota declined to 43.3–49.8%. In the adjacent HAF group, Basidiomycota remained dominant (>99.3%), with Ascomycota proportions similar to MOCK, indicating that the pathogen had not spread to surrounding tissues.

At the genus level (Figure S1H), *Trichoderma* exhibited a dramatic increase in DiR (43.6–48.7%), accompanied by secondary enrichment of *Talaromyces* (up to 12.2%), whereas both genera were nearly undetectable in MOCK and HAF (<0.002%). Host-associated genera, such as *Stereum* and *Tremella*, were significantly reduced in DiR (*Stereum* declined from 49.2% in MOCK to 31.1%), yet remained abundant in HAF, indicating that *Trichoderma*’s competitive advantage was primarily confined to directly infected regions.

At the species level (Figure 2H), *T. lixii*, *T. afroharzianum*, and *T. atrobrunneum* were specifically enriched in DiR and were absent in HAF and MOCK, identifying them as key pathogenic species in *Trichoderma* infections. *T. amestolkiae* also showed a significant increase in DiR but was nearly undetectable in other groups, suggesting a potential synergistic role in enhancing *Trichoderma* infection. Host-associated species, such as *S. hirsutum* and *T. aurantialba*, exhibited a 50–60% reduction in DiR but remained stable in HAF, further supporting the hypothesis that *Trichoderma*’s competitive suppression was localized to infected tissues.

#### 3.1.5. Species Variation Analysis and Biomarker Identification

**Figure 3:**
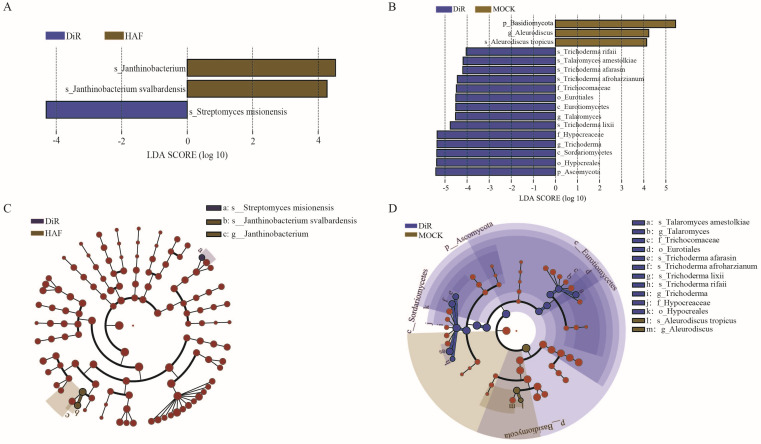
Microbial Biomarker LEfSe Analysis. (**A**,**B**): Bar charts of LDA discrimination scores for bacteria (**A**) and fungi (**B**) based on LEfSe analysis (LDA score ≥ 2 is the screening threshold; different colours represent different groups) [30]. (**C**,**D**): Phylogenetic trees of bacteria (**C**) and fungi (**D**) generated by LEfSe (classification information from phylum to species level is represented from the innermost to the outermost circle; node size is proportional to relative abundance; reddish-brown indicates taxonomic units with no significant differences between groups).

The LEfSe (Linear Discriminant Analysis Effect Size) method [30] was employed to identify statistically significant microbial biomarkers between different groups. The bacterial LEfSe analysis (Figure 3A) revealed that in the HAF group, which was adjacent to the infection site, the bacterial community exhibited specific enrichment patterns. It should be noted that LEfSe analysis may be sensitive to small sample sizes, and thus the identified biomarkers should be interpreted with caution. At the species level, *J. svalbardensis* (LDA = 4.29) and *S. misionensis* (LDA = 4.29) were significantly enriched, with LDA scores far exceeding the threshold (>2). Phylogenetic analysis further indicated that these species had a notably higher relative abundance in the HAF group than in the DiR group. At the phylum level, *J. svalbardensis* and *S. misionensis* belong to Proteobacteria and Actinobacteria, respectively, suggesting that the host in the HAF group may selectively recruit these functional bacterial phyla to establish a microbial defense barrier. At the genus level, the co-enrichment of *Janthinobacterium* and *Streptomyces* was particularly evident. *Janthinobacterium* is well known for producing antimicrobial pigments such as violacein, while *Streptomyces* is renowned for synthesizing broad-spectrum antibiotics like streptomycin. This co-occurrence suggests that these bacteria may help suppress the growth of pathogenic fungi such as *Trichoderma*, thereby contributing to the ecological stability of the boundary region. This finding holds potential significance for food preservation and safety, as microbial competition and natural antagonism could be leveraged to develop novel biocontrol strategies for preventing fungal contamination in food products.

Phylogenetic analysis (Figure 3C) further confirmed the enrichment of *S. misionensis* (node a), *J. svalbardensis* (node b), and the genus *Janthinobacterium* (node c) in the HAF group. The size of the *S. misionensis* node suggests its prominent role in pathogen suppression, likely through antibiotic production. Moreover, *Janthinobacterium* (node c) expanded significantly under *Proteobacteria*, whereas *Streptomyces* (node a) was classified under Actinobacteria, indicating a potential synergistic effect between these functionally distinct bacterial groups in maintaining the stability of the HAF microbial community.

The fungal LEfSe analysis (Figure 3B) revealed that the DiR group (infected tissue) was dominated by *Ascomycota*, with *Trichoderma* (order Hypocreales) and *Talaromyces* (order Eurotiales) as key differential taxa. At the species level, *Trichoderma lixii* (LDA = 4.8), *T. afroharzianum* (LDA = 4.4), and *T. amestolkiae* (LDA = 4.2) were significantly enriched in the DiR group, with LDA scores exceeding the threshold (>2). The phylogenetic tree confirmed that these species had a high relative abundance in the DiR group, highlighting their direct involvement in the pathogenic infection process.

In contrast, the fungal community in the MOCK group (healthy tissue) was dominated by Basidiomycota, with *Aleurodiscus tropicus* (LDA = 4.1) as the most abundant biomarker species. This suggests that *A. tropicus* may play a role in competitive exclusion of fungal pathogens, preventing their colonization. Notably, species from Basidiomycota have been reported to produce antifungal compounds, which could be explored for food preservation applications.

Further phylogenetic analysis (Figure 3D) showed that nodes representing *Ascomycota* in the DiR group, including Hypocreales and Eurotiales, were significantly expanded, while Basidiomycota nodes such as *Aleurodiscus* were dominant in the MOCK group. Although the HAF group was not explicitly highlighted in this analysis, the stark contrast between the DiR and MOCK groups suggests that *Trichoderma*’s pathogenic effect is strictly confined to the infection site. The host’s healthy tissue appears to counteract infection by maintaining a high abundance of Basidiomycota and functional genera like *Aleurodiscus*, which could act as an ecological barrier against fungal invasion.

### 3.2. Amino Acid Targeted Metabolic Analysis

#### 3.2.1. Analysis of Sample Quality Control

This study employed liquid chromatography-tandem mass spectrometry (LC-MS/MS) to quantitatively analyze 94 amino acids and their metabolites (Table S3). The reliability of the experimental data was validated through multidimensional quality control experiments: Total ion chromatograms (TIC) revealed that the chromatographic profiles of all samples were highly consistent (Figure S2A), and the extracted ion chromatogram (XIC) peaks in both positive and negative ion modes showed symmetrical and sharp shapes (Figure S2B,C). The TIC overlay confirmed that the retention time deviation was less than 0.1 min (Figure S2D), ensuring accurate quantification. The Pearson correlation coefficients for QC samples ranged from 0.9992 to 0.9996 (Figure S2E), demonstrating excellent experimental reproducibility. Coefficient of variation (CV) analysis of all samples (including QC) showed that over 80% of metabolites had a CV value less than 0.3 (Figure S2F), meeting metabolomics data quality control standards. This analytical system demonstrated robust data reliability through four-dimensional verification—chromatographic consistency (Figure S2A–D), instrument stability (Figure S2B–D), experimental reproducibility (Figure S2E), and data precision (Figure S2F)—ensuring a solid foundation for subsequent metabolic pathway analysis.

#### 3.2.2. Standard Curve for the Quantification of Amino Acids Metabolites

Table S4 presents the linear equations and correlation coefficients (r) associated with the standard curves employed in the quantitative analysis of 94 amino acid metabolites. The correlation coefficients (r), which range from 0.99029 to 0.99987, demonstrate a robust linear relationship across all standard curves, thereby meeting the established experimental quality criteria. These findings highlight the reliability and precision of the calibration models, affirming their suitability for the subsequent quantification of metabolites in the analyzed samples.

#### 3.2.3. Absolute Quantification of Amino Acids Metabolites

Through absolute quantitative metabolomics analysis (refer to Table S5), notable differences in amino acid metabolism were identified between the brown diseased regions (DiR) and the healthy tissues (MOCK and HAF) of *N. sinensis*. Key metabolites associated with protein synthesis and redox homeostasis were systematically downregulated in the DiR. Specifically, the concentration of O-Phospho-L-Serine in DiR (6.33 × 10^2^ ng/g) was diminished by 17.55-fold and 39.34-fold relative to MOCK (1.11 × 10^4^ ng/g) and HAF (2.49 × 10^4^ ng/g), respectively, indicating a pronounced suppression of phosphorylated amino acid metabolism. Similarly, L-Lysine levels in DiR (1.47 × 10^4^ ng/g) were 10.97- to 16.45-fold lower than those in healthy tissues, suggesting potential disruptions in protein degradation or transport processes. A reduction of 8.62- to 14.43-fold in oxidized glutathione levels (4.54 × 10^3^ vs. 3.91 × 10^4^–6.56 × 10^4^ ng/g) further implied compromised oxidative stress defense mechanisms. Additionally, 12 other metabolites, including L-Tryptophan, Glycine, and Argininosuccinic acid, were significantly reduced, collectively indicating dysregulation of nitrogen metabolism and energy deficits during pathogenesis.

Contrary to the prevalent downregulation observed, metabolites linked to the urea cycle were markedly enriched in DiR. The concentration of 2-aminobutyric acid in DiR (3.33 × 10^4^ ng/g) exhibited an increase of 78.52% and 91.03% compared to MOCK and HAF, respectively, suggesting its potential role as an antioxidant in stress response. Additionally, L-glutamine levels (1.48 × 10^6^ ng/g) rose by 51.57% to 80.30% in DiR, indicating a shift in nitrogen storage forms. The concentrations of L-citrulline and urea increased by 17.69% to 63.48% and 43.78% to 61.00%, respectively, further corroborating the activation of the urea cycle as a response to ammonia toxicity. This metabolic reprogramming may facilitate the maintenance of partial cellular function by modulating intracellular pH and nitrogen balance.

DiR demonstrated distinct metabolic alterations, characterized by the complete absence of L-Cystine, 5-Hydroxy-tryptophan, and 3-Hydroxyhippuric acid, while N,N-Dimethylglycine was uniquely present in DiR, with a detection threshold exceeding 10 ng/g. The absence of Trans-4-Hydroxy-L-Proline in HAF indicates potential early disruptions in proline-related metabolism within adjacent healthy tissues. Additionally, the concentration of Trimethylamine N-Oxide in DiR (33.87 ng/g) was intermediate between that in MOCK (76.90 ng/g) and HAF (7.53 ng/g), possibly indicating alterations in microbial symbiosis. Notably, L-Glutamic acid levels in DiR (2.80 × 10^6^ ng/g) increased by 37.93% compared to HAF but did not fully return to MOCK levels (2.97 × 10^6^ ng/g), suggesting compensatory metabolic adjustments in specific pathways.

#### 3.2.4. Principal Component Analysis

**Figure 4:**
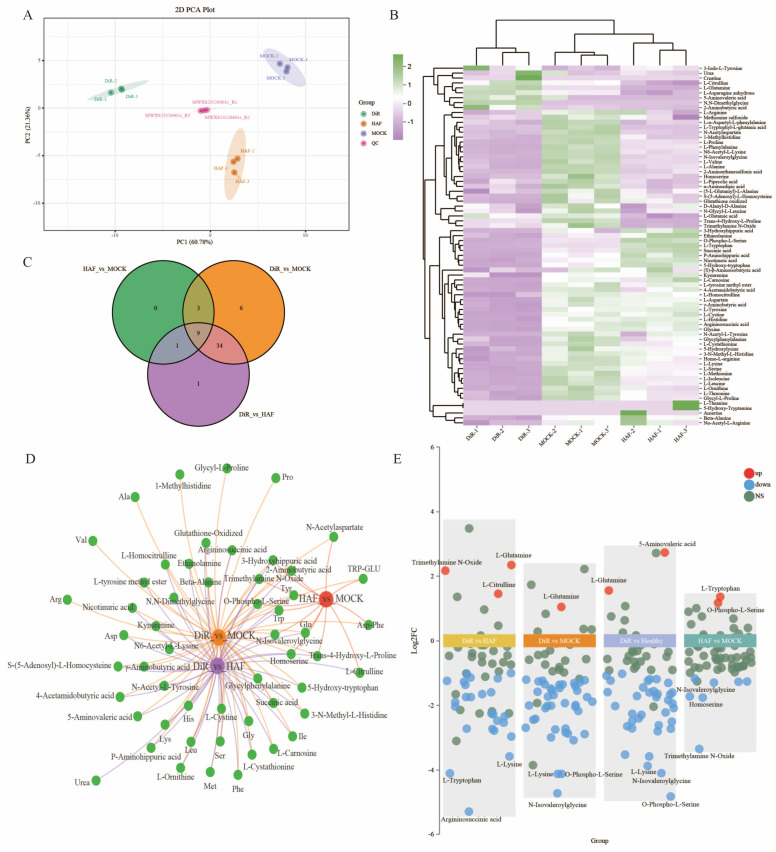
Targeted Amino Acid Metabolomics Analysis. (**A**) PCA score plot (PC1: 60.78%, PC2: 21.36%). (**B**) Clustering heatmap of 73 amino acid metabolites (data Z-score normalized, green: upregulated, purple: downregulated). (**C**) Differential metabolite flower plot. (**D**) Differential metabolites network Venn diagram. (**E**) Multiple group volcano plots (criteria: removal of outliers, log2FC ≥ 1, FDR < 0.05, top 6 metabolites labeled).

Principal Component Analysis (PCA) demonstrated a marked spatial differentiation in the metabolomes of MOCK, DiR, and HAF groups (Figure 4A). The first principal component (PC1), accounting for 60.78% of the variance, and the second principal component (PC2), accounting for 21.36%, collectively elucidated 82.14% of the metabolic variability. The separation along PC1 distinctly differentiated diseased states from healthy ones. Quality control (QC) samples were tightly clustered around the origin, with a technical error margin of less than 5%, thereby confirming the data’s reliability. DiR samples (DIR-1 to DIR-3) showed a significant deviation along the negative axis of PC1, in stark contrast to the MOCK samples, which were highly clustered along the positive PC1 axis. This suggests a global metabolic reprogramming in diseased regions, characterized by disruptions in energy metabolism and redox balance. This observation aligns with the previously noted systemic depletion of amino acid metabolites (Table S5). Although HAF samples (HAF-1 to HAF-3) partially overlapped with MOCK, they exhibited a slight shift along the positive PC1 axis. This indicates that while adjacent tissues maintain essential metabolite homeostasis, they may have undergone adaptive regulatory responses to pathogen stress, forming a progressive metabolic defense gradient.

#### 3.2.5. Cluster Analysis of 73 Quantified Amino Acids Metabolites

The cluster analysis of 73 amino acids and their derivatives (Figure 4B) demonstrated a distinct spatial divergence in the metabolic profiles of healthy tissues (MOCK), diseased regions (DiR), and adjacent healthy tissues (HAF) in *N. sinensis*. The heatmap analysis categorized the metabolites into three primary clusters. Cluster I, characterized by a general downregulation in DiR, included Glutathione oxidized, O-Phospho-L-Serine, and L-Tryptophan, which exhibited significantly reduced levels compared to MOCK (Table S5). This finding suggests a substantial impairment of antioxidant defense mechanisms and amino acid phosphorylation pathways in the affected region. Cluster II comprised metabolites specifically upregulated in DiR, such as Urea, L-Citrulline, and 2-Aminobutyric acid, with urea levels increasing by 43.78% and 61.00% compared to MOCK and HAF, respectively. This indicates a pronounced metabolic shift toward the urea cycle for nitrogen detoxification. Cluster III reflected transitional regulation in HAF, where metabolites like L-Glutamine and γ-Aminobutyric acid were maintained at levels comparable to MOCK, while others, such as Trans-4-Hydroxy-L-Proline, were absent in HAF, implying a selective metabolic suppression as a preadaptive response to pathogen pressure.

The depletion of Cluster I metabolites was closely linked to metabolic collapse in DiR. For example, oxidized glutathione levels in DiR (4.54 × 10^3^ ng/g) dropped by 93.09% compared to MOCK, impairing ROS scavenging. Additionally, a significant reduction in O-Phospho-L-Serine (DiR vs. MOCK: 6.33 × 10^2^ vs. 1.11 × 10^4^ ng/g) likely disrupted the serine-glycine-one-carbon metabolic axis, worsening energy metabolism dysregulation. Conversely, the accumulation of urea cycle intermediates in Cluster II, such as urea and L-citrulline, indicated increased nitrogen detoxification demand in DiR. Notably, L-citrulline levels (3.11 × 10^4^ ng/g) rose by 63.48% in DiR compared to HAF, suggesting abnormal activation of the arginine metabolism pathway. Furthermore, the absence of HAF-specific metabolites in Cluster III (e.g., N,N-Dimethylglycine) and the loss of MOCK-specific metabolites (e.g., L-Cystine) supported the notion of targeted disruption of sulfur metabolism due to pathogen infection.

#### 3.2.6. Screening of Differential Amino Acid Metabolites

Differential metabolite screening (fold change ≥ 2 or ≤0.5) revealed significant metabolic disruptions in diseased *N. sinensis* tissues, with 45, 52, and 52 metabolites differing from HAF, MOCK, and the healthy group, respectively. Most changes (82–92%) were downregulations, indicating widespread suppression of amino acid pathways, especially those related to phosphorylation and redox balance (Tables S7–S9). Differences between HAF and MOCK were minimal, with only 13 metabolites differing (3 upregulated, 10 downregulated, Table S10). A flower Venn diagram (Figure 4C) identified nine core metabolites shared across all groups, including significant decreases in O-Phospho-L-Serine and L-tryptophan, indicating a collapse in the serine-glycine axis and indole biosynthesis. Six metabolites were unique to the DiR_vs_MOCK group, while HAF_vs_MOCK had none, highlighting infection-induced metabolic destabilization. The network Venn diagram analysis (Figure 4D) provided insights into functional clustering. The consistent downregulation of oxidized glutathione across all DiR comparisons suggests impaired reactive oxygen species (ROS) detoxification. Alterations in S-(5-adenosyl)-L-homocysteine levels indicate disruptions in the methylation pathway. The distinct expression patterns of urea and β-alanine in the DiR_vs_HAF comparison suggest region-specific nitrogen detoxification processes. Notable fluctuations in aromatic amino acids, such as L-tyrosine and L-phenylalanine, imply disruptions in secondary metabolism, which may exacerbate disease-related browning. Collectively, these findings underscore pathogen-induced metabolic reprogramming in DiR, characterized by energy deficits, amino acid dysregulation, and oxidative stress imbalance.

A metabolomic analysis using volcano plot criteria (log_2_FC ≥ 1, *p* < 0.05, excluding Inf/-Inf outliers) was performed on diseased brown regions (DiR), adjacent healthy tissues (HAF), the healthy control group (MOCK), and a combined healthy group (HAF + MOCK). This revealed significant metabolic changes, especially in amino acid metabolism, with the DiR group showing the most notable alterations, indicating significant metabolic reprogramming (Figure 4E). In the DiR_vs_HAF comparison, changes were mainly linked to nitrogen metabolism and oxidative stress. L-glutamine and trimethylamine N-oxide were upregulated, suggesting nitrogen metabolism remodeling and microbial disturbances, while argininosuccinic acid, L-tryptophan, and L-lysine were downregulated, indicating potential disruptions in the urea cycle and tryptophan metabolism related to oxidative stress. In the DiR_vs_MOCK comparison, L-glutamine was consistently upregulated, while N-isovaleroylglycine and O-phospho-L-serine were significantly downregulated, indicating suppressed branched-chain amino acid metabolism and impaired phospholipid synthesis, which may lead to metabolic stress and energy depletion. In the DiR_vs_Healthy comparison, 5-aminovaleric acid increased, suggesting microbial metabolic changes, while the downregulation of O-phospho-L-serine, N-isovaleroylglycine, and L-lysine points to a broad metabolic imbalance affecting amino acid biosynthesis and cellular homeostasis. Adjacent healthy tissues (HAF_vs_MOCK) showed partial metabolic compensation. The upregulation of L-tryptophan and O-phospho-L-serine suggests their role in antioxidant defense, while the significant downregulation of trimethylamine N-oxide indicates microbial metabolic disruptions. These findings highlight that the diseased tissue undergoes metabolic reprogramming marked by nitrogen metabolism issues, oxidative stress, and amino acid metabolism imbalances, leading to metabolic network instability.

**Figure 5:**
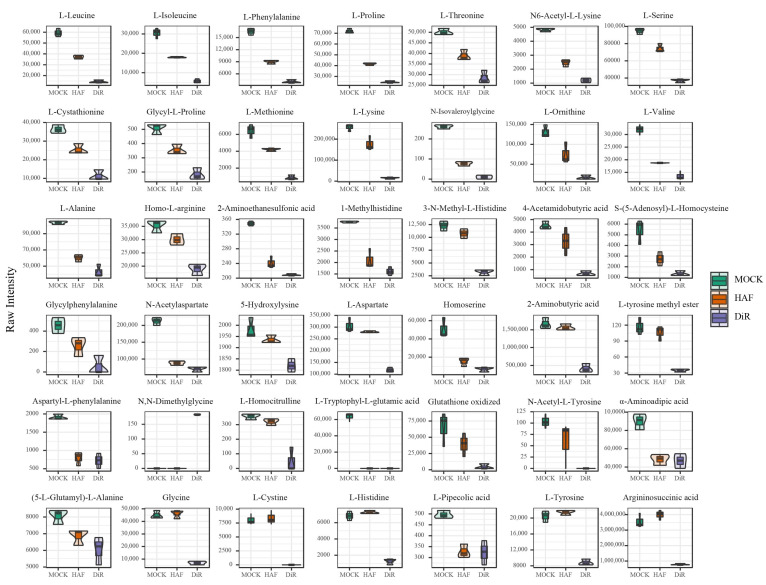
Expression Distribution of Differential Metabolites. Violin plots of differential amino acid metabolite expression (*x*-axis: groups, *y*-axis: log10 normalized intensity), showing the top 50 metabolites with VIP >1.5 and FDR < 0.01.

Violin plot analysis (Figure 5) and differential metabolite screening (VIP > 1, *p* < 0.05, Table S11) revealed significant metabolic changes across tissue samples. Amino acid metabolism, especially branched-chain and aromatic amino acids, was systematically downregulated. L-leucine levels decreased progressively in the MOCK, HAF, and DiR groups, with mean concentrations of 5.94 × 10^4^ ng/g, 3.71 × 10^4^ ng/g, and 1.44 × 10^4^ ng/g, respectively (VIP = 1.27, *p* = 1.92 × 10^−6^), indicating severe protein biosynthesis impairment in the DiR group. L-Phenylalanine levels dropped by 75.39%, from 1.65 × 10^4^ ng/g in the MOCK group to 4.06 × 10^3^ ng/g in the DiR group (VIP = 1.27, *p* = 8.40 × 10^−7^), suggesting disease-related carbon skeleton redistribution. Oxidative stress-related metabolites showed a polarized response. In the DiR group, levels of oxidized glutathione (GSSG) markedly declined to 4.54 × 10^3^ ng/g, indicating a 93.09% reduction relative to the MOCK group (VIP = 1.10, *p* = 0.019). This finding suggests a potential compromise in antioxidant defense mechanisms. In contrast, levels of N,N-Dimethylglycine in the DiR group significantly rose to 182.75 ng/g (VIP = 1.11, *p* = 3.54 × 10^−12^), while remaining undetectable in both the MOCK and HAF groups. Given its function as a methyl donor and osmoprotectant, this increase likely represents a metabolic compensation mechanism designed to counteract oxidative stress, thereby underscoring a dynamic imbalance in redox homeostasis. Additionally, spatial metabolic gradients revealed early response mechanisms in adjacent healthy tissues (HAF). Argininosuccinic acid demonstrated a transient elevation in the HAF group (3.99 × 10^6^ ng/g, representing a 12.28% increase compared to the MOCK group, VIP = 1.01, *p* = 3.67 × 10^−5^), before subsequently decreasing to 7.86 × 10^5^ ng/g in the DiR group. This pattern suggests a localized activation of the nitric oxide synthesis pathway at the disease margin, potentially serving to delay disease progression. In contrast, 5-Hydroxylysine levels remained stable in the HAF group (1.94 × 10^3^ ng/g compared to MOCK 1.98 × 10^3^ ng/g) but decreased in the DiR group (1.82 × 10^3^ ng/g, VIP = 1.17, *p* = 0.003), indicating possible disruptions in collagen modification specifically within the core infection site.

#### 3.2.7. Analysis of Metabolic Pathways of Different Amino Acid Metabolites Based on the KEGG Database

**Figure 6:**
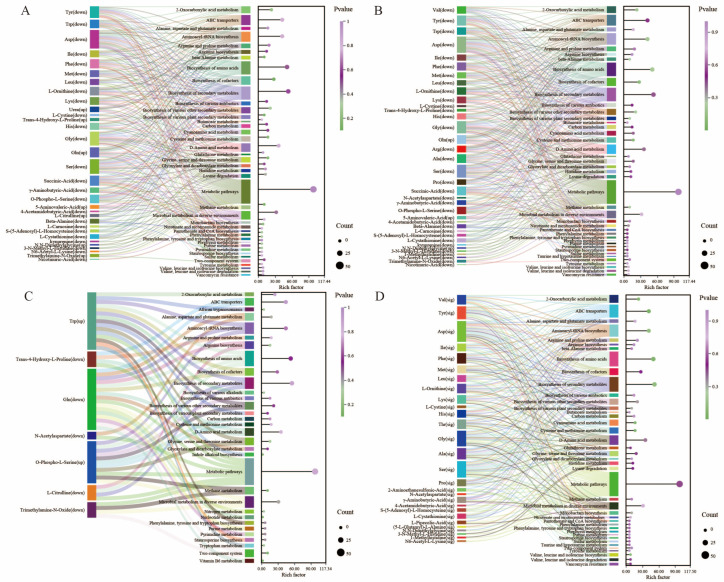
Pathway Enrichment Analysis of Differential Metabolites. Sankey bubble plot showing metabolite-pathway associations (line width proportional to metabolite count), with the right-side KEGG enrichment bubble plot (Rich factor = number of differential metabolites/total metabolites in the pathway), point size indicating the number of differential metabolites, and color gradient representing −log10(*p*) values. (**A**–**C**) Group comparisons (DiR vs. HAF, DiR vs. MOCK, HAF vs. MOCK). (**D**) Three-group combined analysis.

Based on KEGG pathway enrichment analysis (Figure 6A–C), amino acid metabolism exhibited clear spatial heterogeneity among diseased tissues (DiR), adjacent healthy tissues (HAF), and healthy controls (MOCK) in *N. sinensis*. In the DiR vs. HAF comparison, 24 metabolites associated with amino acid biosynthesis, including S-(5-adenosyl)-L-homocysteine, O-phospho-L-serine, tyrosine, tryptophan, and aspartate, were systematically downregulated (metabolic frequency 51.06%, *p* = 0.460). Similarly, within the D-amino acid metabolism pathway, 17 metabolites were identified (metabolic frequency 31.17%, *p* = 0.759), with histidine, glycine, and proline downregulated, whereas 5-aminovaleric acid was upregulated. These changes suggest that D-amino acid-related metabolism may be involved in modulating cell signaling or stress responses associated with pathogen defense (Figure 6A). Moreover, changes in ABC transporters (metabolic frequency 42.55%, *p* = 0.761), together with the perturbation of L-cystine, indicate a potential role of transmembrane transport in resource redistribution. The downregulation of phenylalanine and leucine in pathways related to secondary metabolite biosynthesis (metabolic frequency 53.19%, *p* = 0.627) further suggests a shift in metabolic resource allocation under infection stress, which may contribute to host defense responses and disease progression.

The comparison between DiR and MOCK conditions revealed a dual pattern characterized by metabolic collapse and stress-induced compensatory mechanisms. Metabolic disturbances in the DiR condition were more pronounced, as evidenced by a widespread suppression in the biosynthesis of 11 essential amino acids, including S-(5-adenosyl)-L-homocysteine, O-phospho-L-serine, valine, and tyrosine, with a metabolic frequency of 51.06% (*p* = 0.221). Concurrently, within the arginine and proline metabolic pathways, there was a significant upregulation of 5-aminovaleric acid (*p* = 0.766), suggesting a metabolic adaptation that favors polyamine synthesis (Figure 6B). Additionally, the coordinated suppression of branched-chain amino acids (BCAAs) within the 2-oxocarboxylic acid metabolism, with a metabolic frequency of 23.40% (*p* = 0.195), alongside the depletion of tricarboxylic acid (TCA) cycle intermediates such as succinic acid and γ-aminobutyric acid, further exacerbates the energy crisis. Notably, L-glutamine exhibited a distinctive upregulation within the ABC transporter pathway, achieving a concentration of 1.48 × 10^6^ ng/g in DiR, as opposed to 7.15 × 10^5^ ng/g in MOCK. This upregulation may facilitate the activity of the TCA cycle to maintain energy production. Nonetheless, this metabolic adjustment was accompanied by a disruption of the urea cycle, as indicated by a significant reduction in argininosuccinic acid (Log_2_FC ≤ −2.17), and a breakdown of the antioxidant defense system, evidenced by the depletion of oxidized glutathione.

The HAF vs. MOCK comparison revealed an early-stage metabolic pre-adaptation in the tissue adjacent to infection sites. Despite appearing phenotypically healthy, HAF exhibited notable metabolic disturbances (Figure 6C). Specifically, biosynthesis of amino acids showed significant suppression of 24 metabolites (e.g., tyrosine, tryptophan, and histidine; metabolic frequency 51.06%, *p* = 0.234), resembling the stress response observed in DiR. Likewise, D-amino acid metabolism showed an upregulation of 5-aminovaleric acid (metabolic frequency 36.17%, *p* = 0.524), suggesting an early metabolic shift to counteract oxidative stress. The suppression of biosynthesis of secondary metabolites (e.g., phenylalanine and leucine; metabolic frequency 53.19%, *p* = 0.381) alongside the activation of ABC transporters (metabolic frequency 42.55%) suggests a metabolic strategy aimed at restricting pathogen spread by enhancing transport efficiency. However, the drastic downregulation of trimethylamine N-oxide (Log_2_FC = −3.35) in HAF implies microbial dysbiosis, potentially compromising its metabolic resilience.

The MOCK-HAF-DiR metabolic cascade analysis unveiled a spatially structured gradient of metabolic reprogramming, which was highly correlated with disease progression (Figure 6D). Biosynthesis of amino acids was significantly enriched (metabolic frequency = 0.50, *p* = 0.071), encompassing 24 key differential metabolites (e.g., L-leucine and L-phenylalanine). Notably, BCAAs declined by 50–75% in DiR compared to MOCK (e.g., L-leucine), suggesting a collapse in protein synthesis capacity. ABC transporters (metabolic frequency = 41.67%, *p* = 0.045) covered 20 metabolites (e.g., L-cystine and glycine), with significant downregulation (e.g., glycine), possibly indicating impaired transmembrane transport. A key observation was the transitional metabolic response in HAF within the arginine and proline metabolism pathway. L-ornithine levels increased by 12.5% in HAF compared to MOCK but dropped sharply in DiR, suggesting that nitric oxide synthesis was transiently activated in adjacent tissues as a defense mechanism. Furthermore, within cysteine and methionine metabolism (metabolic frequency = 16.67%, *p* = 0.071), S-(5-Adenosyl)-L-Homocysteine declined by 67% in DiR relative to MOCK, whereas γ-aminobutyric acid accumulated, illustrating a disruption of oxidative stress regulation and neurotransmitter balance.

Our findings highlight a systemic collapse of amino acid biosynthesis in diseased tissues, with 24 key metabolites (e.g., L-leucine and L-phenylalanine) displaying a 50–75% reduction relative to MOCK (*p* = 0.071), directly impairing protein synthesis. Concurrently, ABC transporter dysfunction (metabolic frequency 41.67%) revealed a widespread inactivation of transmembrane energy-material exchange. A striking metabolic gradient emerged in the arginine and proline metabolism pathway, where L-ornithine was significantly upregulated in HAF but drastically depleted in DiR, indicating that transient activation of nitric oxide synthesis might serve as a critical defensive barrier in adjacent tissues. Furthermore, within the metabolic pathways of cysteine and methionine, the pronounced downregulation of S-(5-adenosyl)-L-homocysteine coupled with the abnormal accumulation of γ-aminobutyric acid outlines a framework indicative of oxidative stress imbalance and neurotransmitter dysregulation. This spatially heterogeneous metabolic reprogramming—from pre-adaptive compensation in HAF to a complete metabolic collapse in DiR—highlights the hierarchical disintegration of metabolic networks during fungal pathogenesis. The suppression of primary metabolism initiates a reallocation of resources towards secondary metabolism. Concurrently, the upregulation of 5-aminovaleric acid and the selective accumulation of glutamine suggest a dynamic metabolic trade-off between host–pathogen interaction and the mitigation of an energy crisis.

## 4. Discussion

In this study, we utilized ONT third-generation sequencing alongside targeted amino acid metabolomics to systematically examine the response mechanisms of the edible and medicinal fungus *N. sinensis* to infection by opportunistic pathogenic fungi of the *Trichoderma*. It is important to note that this study is correlational in nature; while *Trichoderma* species were found to be enriched in diseased tissues, causality has not been established as Koch’s postulates were not performed. Thus, the following discussion focuses on observed associations and potential mechanisms that warrant further experimental validation. Through a comprehensive analysis of the microbial community structure, encompassing both bacteria and fungi, as well as the host amino acid metabolic networks across different regions of the fruiting body—namely, the healthy control (MOCK), adjacent healthy area (HAF), and diseased core (DiR)—our results show that the presence of *Trichoderma* is associated with a significant disruption of the stable symbiotic microbiota equilibrium within the host, alongside a marked shift in microbial community composition, which we refer to as a “hijacking-like” phenomenon. However, whether this shift is directly caused by *Trichoderma* or represents a consequence of host tissue deterioration remains to be determined through functional experiments. During the spread of the pathogen, the microbial community transitioned from a stable symbiotic state in healthy conditions to a disordered state in diseased conditions. Concurrently, the host’s amino acid metabolic pathways underwent systematic remodeling, marked by significant imbalances in key metabolites and metabolic pathways, including the biosynthesis of amino acids, D-amino acid metabolism, ABC transporters, and the biosynthesis of secondary metabolites. This intricate interplay between shifts in the microbial community and host metabolism offers a novel perspective for understanding the pathogenic mechanisms of *Trichoderma* and suggests avenues for the development of precise intervention strategies.

The strength of this study lies in the integration of microbiome and metabolome data, which preliminarily revealed the potential link between the two. For example, the enrichment of *Proteiniborus* (related to cellulose degradation) in the DiR region may be associated with the release of host structural amino acids. The enrichment of *Janthinobacterium*/*Streptomyces* in the HAF region may affect the local metabolic environment through secretions, thereby inhibiting the metabolic activity of *Trichoderma*. However, it should be noted that this study still has some limitations. First, the study design was based on sampling at specific time points, failing to fully capture the continuous dynamic changes during pathogen infection. Future time-series studies will help to better understand the causal relationships in disease progression. Second, targeted metabolomics focused only on amino acids and their derivatives, failing to cover other potentially important metabolic pathways (such as carbohydrates, lipids, secondary metabolites). Non-targeted metabolomics or multi-platform metabolomics analyses will provide a more comprehensive metabolic landscape. Finally, the correlations revealed in this study need to be further verified through functional experiments (such as isolation and re-inoculation of key microbes, host gene function validation, metabolic flux analysis, etc.) to establish causal relationships. One limitation of this study is the rela2+tively small sample size (*n* = 3 per group), which may limit statistical power and increase uncertainty in the microbiome analysis. Although the results show consistent trends across the replicate groups, these findings should be interpreted with caution. Future studies with larger sample sizes are needed to validate these findings.

In the analysis of microbial community α-diversity, a distinct gradient was observed in bacterial communities across the MOCK, HAF, and DiR samples. The MOCK group demonstrated the highest levels of richness (ACE > 268) and evenness (Shannon > 4.24), indicative of a long-term stable symbiotic relationship between the host and its microbial inhabitants. Conversely, the DiR group exhibited a marked reduction in both ACE and Shannon indices, suggesting that pathogen infection resulted in a loss of community diversity and a disruption of ecological equilibrium. This phenomenon is consistent with the community disorder effect reported in studies of other host–pathogen systems, Bulgari et al. (2014) [31]. Furthermore, the HAF region, serving as a transitional zone between health and disease, exhibited an intermediate state between MOCK and DiR, suggesting that local ecological changes had occurred in this area. Whether these changes represent active stress responses of the fungus or passive consequences of tissue degradation requires further investigation. In comparison to bacterial communities, fungal communities exhibited more pronounced alterations. Specifically, within the DiR group, the Shannon and Simpson indices were significantly reduced compared to the MOCK group, while the relative abundance of pathogenic *Trichoderma* markedly increased within the diseased core area (43.6–48.7%), remaining nearly undetectable in the MOCK and HAF groups (<0.2%). This observation aligns with the findings of Zhang et al. (2021) [32], which reported a decline in fungal community diversity and a simplification of co-occurrence networks following pathogen invasion. Moreover, it underscores the critical role of host symbiotic fungi in sustaining ecological equilibrium. Further β-diversity analysis demonstrated significant distinctions between bacterial and fungal communities, with the community structure transitioning from stable symbiosis in the healthy state to disorder in the diseased state as the pathogen proliferated. Nevertheless, given the differences between plants and fungi in terms of physiological structure and stress response mechanisms, such comparisons serve primarily to provide a reference framework; the specific mechanisms still require further validation in fungal systems.

Delving deeper into the composition of bacterial communities, we found that *Proteobacteria* dominated in all sample groups, with an average relative abundance exceeding 85.6% in the MOCK group but dropping to 68.7% in the DiR group. Conversely, Firmicutes reached an abundance of 22.6% in the DiR3 group, significantly higher than in other groups (typically ranging from 0.8% to 6.8%), suggesting that pathogen infection may induce abnormal proliferation of certain Gram-positive bacteria, closely related to the host’s metabolic dysregulation. Meanwhile, *Actinobacteriota* had a low abundance in the MOCK group (below 3.4%) but was significantly upregulated to 5.6% in the DiR2 group, implying that this phylum of bacteria may play a positive role in stress response. Notably, the proportion of unclassified microorganisms in the DiR group was as high as 29.7%, indicating that pathogen infection may trigger abnormal proliferation of some microorganisms with unknown functions, further complicating community structure. Previous studies have shown that pathogen infection not only directly damages plant health but also significantly reshapes the structure and composition of endophytic bacterial communities [33]. This reshaping process may reflect the activation of host defense mechanisms; that is, hosts under pathogen stress may regulate their endophytic microbial communities in an attempt to restore ecological homeostasis. Moreover, pathogen invasion may also promote the proliferation of certain bacterial genera that may play dual roles in pathogen spread and host defense responses [34]. Overall, these results not only reveal the profound impact of pathogen infection on microbial community diversity and structure but also provide new insights into plant-microbe interactions and their functions in disease control. Additionally, in the HAF region, the significant enrichment of *Janthinobacterium* and *Streptomyces* in bacterial communities offers the possibility of constructing a local “defensive barrier.” These two genera are renowned for producing a variety of antimicrobial/antifungal secondary metabolites [35,36] and may inhibit the spread of *Trichoderma* by modulating the local metabolic environment [37]. This pathogen-induced restructuring of bacterial communities suggests that the host may recruit specific beneficial microbial communities to combat disease and maintain ecological homeostasis [38].

In response to the dynamic alterations observed in bacterial communities, the reorganization of fungal communities was even more pronounced. In the healthy state (MOCK group), *Basidiomycota* were nearly exclusively dominant, comprising 99.7–99.9% of the community, which suggests a robust symbiotic relationship between the host and *Basidiomycota*. Conversely, in the diseased core area (DiR group), the abundance of *Basidiomycota* significantly decreased to 43.3–49.8%, while the proportion of *Ascomycota* increased markedly to 53.4%, 56.6%, and 50.1%, respectively. This shift fully illustrates the profound impact of *Trichoderma* invasion on the fungal community structure. In the DiR group, *Trichoderma* demonstrated significant enrichment, particularly with two pathogenic species, *T. lixii* (abundance ranging from 10.7% to 12.2%) and *T. afroharzianum* (abundance ranging from 5.1% to 5.9%), being notably prominent. Concurrently, the abundance of host symbiotic fungi, such as *S. hirsutum*, decreased by 50% to 60% in the DiR region, while remaining relatively stable in the HAF area. This suggests that *Trichoderma* may directly inhibit the growth of host symbiotic fungi through the secretion of secondary metabolites with antimicrobial and antifungal properties [39]. Although *Trichoderma* are widely employed as biocontrol agents in agricultural production due to their recognized antagonistic and parasitic capabilities [40], our study reveals that under specific conditions, this genus can also become pathogenic, thereby adversely affecting host health [41].

Through targeted amino acid metabolomics analysis, we observed marked changes in the metabolic profile of *N. sinensis* tissues in association with *Trichoderma* colonization. A comparative analysis of diseased tissue (DiR) and adjacent healthy tissue (HAF) revealed a systematic downregulation of 24 metabolites associated with amino acid synthesis pathways, including S-(5-adenosyl)-L-homocysteine, O-phosphorylated L-serine and tyrosine, indicating that protein synthesis was markedly inhibited. These findings must be interpreted with caution. As described in the sample characterization, the DiR tissue exhibited “soft rot” and a “decayed texture,” indicating that extensive cell death and necrosis had occurred in this region. Therefore, the observed downregulation of amino acid synthesis and protein metabolism may, at least in part, reflect a collapse of cellular metabolic function secondary to tissue necrosis, rather than a coordinated adaptive response initiated by living host cells. Distinguishing between active host responses and the passive consequences of cell death requires time-series experiments as well as single-cell or spatial metabolomics analyses. Concurrently, amino acids involved in D-amino acid metabolic pathways, such as histidine, glycine and proline, also exhibited significant decreases. Conversely, the upregulation of 5-aminopentanoic acid raises the possibility that certain metabolic changes may be associated with stress responses, possibly through the regulation of cellular stress responses or signal transduction. However, whether these changes represent active defense mechanisms of the host or are simply byproducts of tissue degradation remains to be determined. Functional validation through controlled inoculation experiments and metabolic flux analysis will be necessary to test these hypotheses. Although previous studies have shown that D-amino acids (such as D-serine and D-aspartic acid) are synthesized by serine isomerases and degraded by D-amino acid oxidases in mammals, participating in the regulation of neurotransmission [42], the role of this pathway in fungal hosts remains to be further validated. Furthermore, significant changes in the ABC transporter pathway support the hypothesis that host cells reallocate resources by regulating transmembrane transport. The downregulation of phenylalanine and leucine in secondary metabolite synthesis pathways may indicate that the affected region limits pathogen proliferation by suppressing primary metabolism and reallocating resources to defence-related secondary metabolism [43]. Further research suggests that certain pathogens impair the host’s energy supply and defence mechanisms by influencing the metabolism of branched-chain amino acids, such as leucine [44].

Subsequent comparative analysis revealed that the metabolic changes in the DiR group were markedly more pronounced than those in the MOCK group, characterized by a dual phenomenon of metabolic collapse and stress compensation. In the DiR group, there was a general downregulation of eleven essential amino acids within the Biosynthesis of Amino Acids pathway, indicating a significant disruption in protein synthesis functionality. Simultaneously, the notable upregulation of 5-aminovaleric acid within the Arginine and Proline Metabolism pathway suggested that the host was attempting to mitigate nitrogen metabolism dysregulation by reallocating resources towards polyamine synthesis. This observation was strongly correlated with the phenotypes of tissue browning and soft rot, which reflect abnormal host signal transduction and the failure of the oxidative stress defense system following pathogen infection. Such findings are consistent with the pathogen’s strategy of commandeering amino acid nutrients to facilitate immune evasion [45]. Furthermore, the reduction in branched-chain amino acids (BCAAs) within the DiR region ranged from 50% to 75%, accompanied by a decrease in tricarboxylic acid (TCA) cycle intermediates, such as succinic acid and γ-aminobutyric acid, thereby intensifying the energy deficit. Notably, the specific upregulation of L-glutamine within the ABC transporters pathway, while beneficial for sustaining the energy supply of the TCA cycle, resulted in the inhibition of the urea cycle and a collapse of the antioxidant system, thus aggravating the host’s metabolic imbalance. The role of the urea cycle in fungal nitrogen metabolism has been elucidated in multiple systems. In the yeast *Candida guilliermondii*, urea cycle enzymes (including arginase and ornithine carbamoyltransferase) are regulated by the availability of arginine and ammonium ions [46]. In arbuscular mycorrhizal fungi (such as *Rhizophagus irregularis*), where arginine serves as a key nitrogen transport compound, the anabolic and catabolic pathways of the urea cycle are spatially separated but functionally highly synchronized [47]. Under nitrogen stress, fungi (such as *Mortierella alpina*) reallocate resources by redirecting amino acid biosynthetic fluxes and recovering nitrogen from protein degradation [48]. These observations suggest that fungal urea cycle activity is closely related to nitrogen status and stress adaptation. In this study, the upregulation of L-glutamine (a key nitrogen carrier) may reflect an attempt to recover nitrogen or maintain tricarboxylic acid cycle intermediates under conditions of extensive amino acid degradation. However, whether this pattern represents an adaptive stress response or a nonspecific consequence of cellular dysfunction cannot be determined from the current observational data.

Furthermore, the metabolic profile of the adjacent healthy area (HAF) was found to be intermediate between MOCK and DiR, exhibiting alterations in key metabolite levels, such as argininosuccinate and tryptophan. This observation suggests that this region had already commenced early metabolic defense mechanisms or adaptive adjustments, underscoring the pivotal role of amino acid metabolism in the stress response of *N. sinensis* to *Trichoderma* colonization [17,49,50]. However, it should be reiterated that all findings presented in this paper are correlational in nature. Future studies must incorporate controlled inoculation, time-series sampling, and functional validation, which are essential for establishing causality and ultimately determining whether *Trichoderma* acts as a primary pathogen or a secondary colonizer in this system.

## 5. Conclusions

This study combined ONT long-read sequencing with targeted metabolomics to comprehensively elucidate the effects of *Trichoderma* infection on the microbial community structure and amino acid metabolome of *N. sinensis*, with a particular focus on spatial heterogeneity across different tissue regions. The results indicate that *Trichoderma* infection leads to a significant reduction in microbial diversity within the lesion core (DiR), whilst bacterial diversity decreases progressively along a gradient (MOCK > HAF > DiR). In the DiR region, the fungal community structure undergoes significant changes, with *T. lixii* and *T. afroharzianum* becoming dominant taxa. Concurrently, the metabolic pathways of *N. sinensis* were significantly altered, including the inhibition of amino acid biosynthesis and antioxidant-related processes, as well as changes in nitrogen metabolism-related pathways. In the adjacent healthy area (HAF), markedly different microbial and metabolic patterns were observed, including the enrichment of *Janthinobacterium* and *Streptomyces*, as well as alterations in specific amino acid-related metabolic pathways. These patterns may reflect local ecological changes associated with the infection state rather than active recruitment by the fungus and suggest a potential link between microbial composition within fungal tissues and environmental adaptation. Overall, these findings deepen our understanding of the interactions between edible fungi, their associated microbial communities, and pathogenic fungi, highlighting the complexity of fungal–microbiome–pathogen interactions in cultivation systems. Furthermore, the study identified several candidate microbial taxa and metabolites that may serve as potential biomarkers for disease-related states. However, further functional validation is required to determine their roles in disease progression and in the stress responses of *N. sinensis*. This study lays a solid theoretical foundation for the development of precise and sustainable biological control strategies for fungal diseases through microbiome regulation or metabolic intervention.

## Data Availability

The raw 16S rRNA and ITS sequencing datasets generated in this study have been made publicly available in the NCBI Sequence Read Archive (SRA) under BioProject accession number PRJNA1457924. The targeted metabolomics data generated in this study are available from the corresponding author upon reasonable request.

## Supplementary Materials

The following supporting information can be downloaded at: https://www.mdpi.com/article/10.3390/foods15111912/s1.
