# Supplementary figures and images for: Oxford Nanopore Technologies Sequencing and Targeted Amino Acid Metabolomics Reveal Spatially Segregated Microbial Hijacking and Metabolic Collapse During Trichoderma Infection of Golden Ear Mushroom
Source: Foods. 2026 May 28;15(11):1912. doi: 10.3390/foods15111912 (PMC13256848; doi:10.3390/foods15111912)

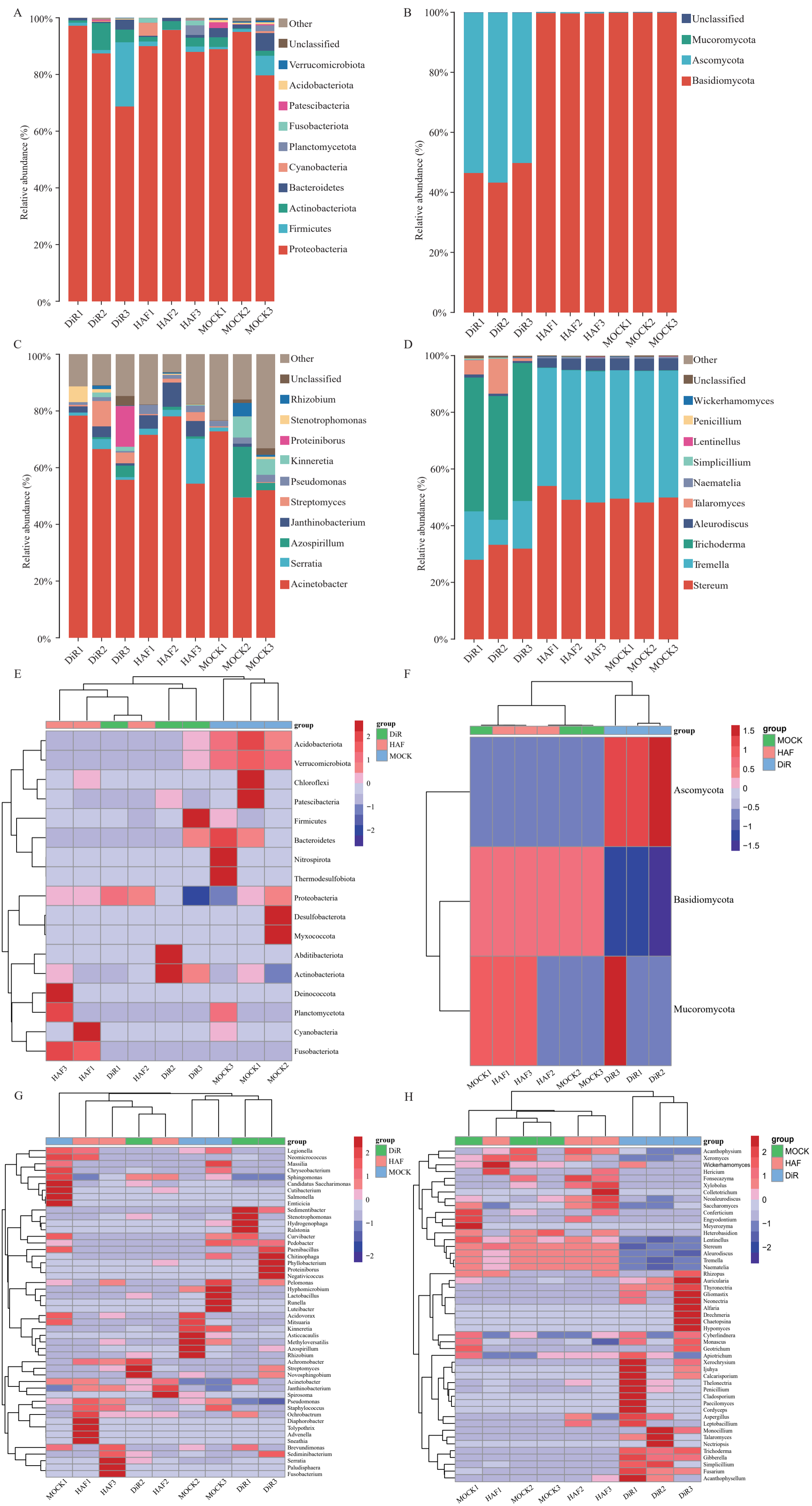

Supplement: Supplementary file 1 [file foods-15-01912-s001.zip › Figure S1A-H.pdf]

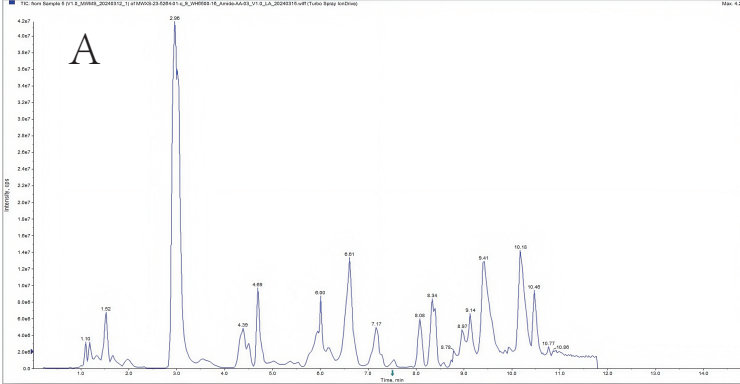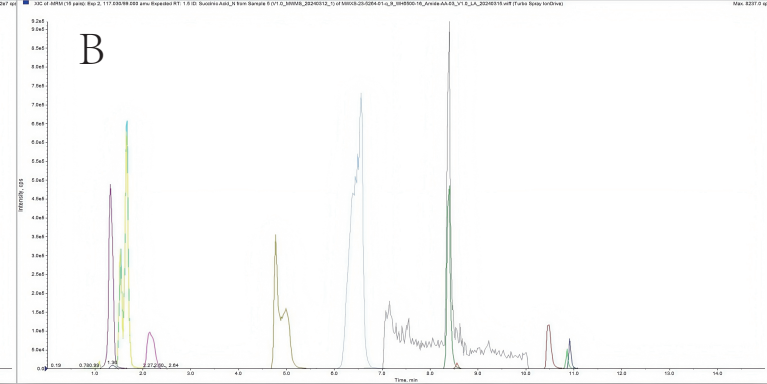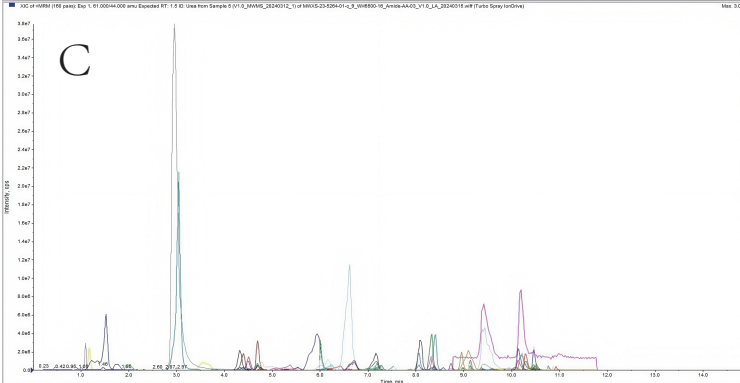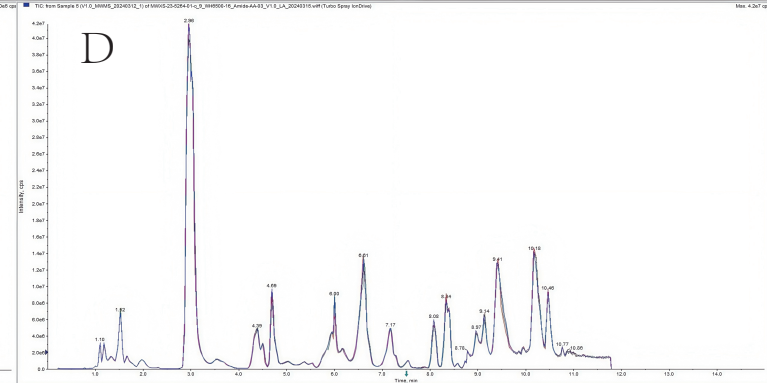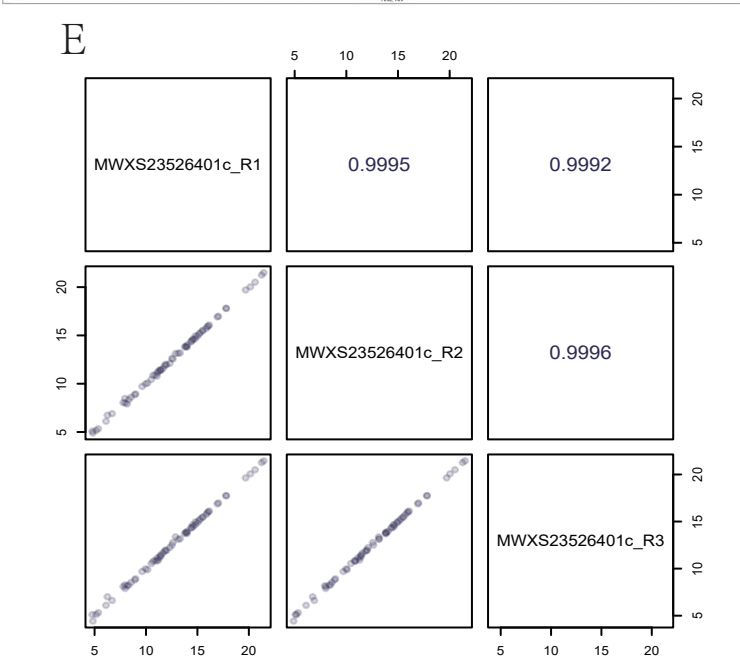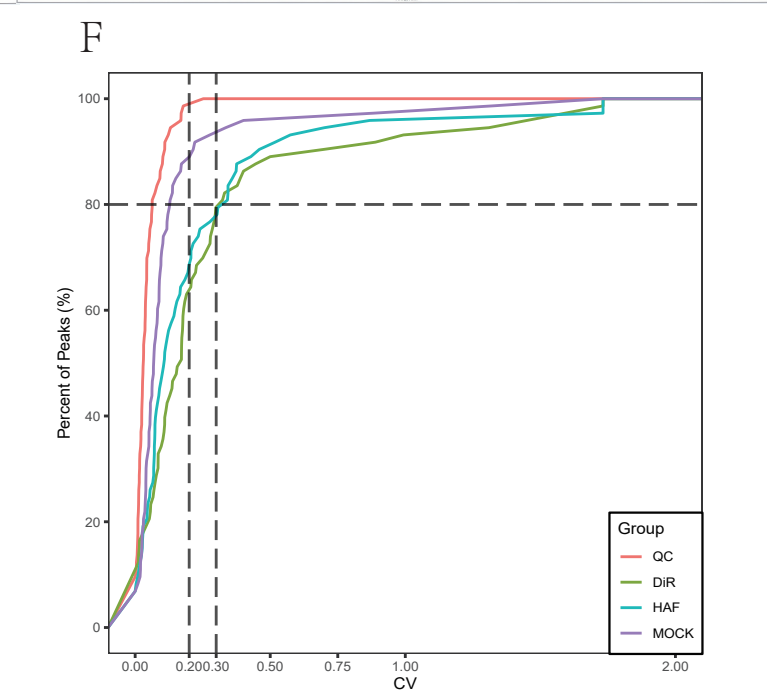

Supplement: Supplementary file 1 [file foods-15-01912-s001.zip › Figure S2A-F.pdf]
